# Data Parameters From Participatory Surveillance Systems in Human, Animal, and Environmental Health From Around the Globe: Descriptive Analysis

**DOI:** 10.2196/55356

**Published:** 2025-03-26

**Authors:** Carrie McNeil, Nomita Divi, Charles Thomas Bargeron IV, Andrea Capobianco Dondona, Kacey C Ernst, Angela S Gupta, Olukayode Fasominu, Lucy Keatts, Terra Kelly, Onicio B Leal Neto, May O Lwin, Mvuyo Makhasi, Eric Beda Mutagahywa, Diego Montecino-Latorre, Sarah Olson, Pranav S Pandit, Daniela Paolotti, Matt C Parker, Muhammad Haiman Samad, Kara Sewalk, Anita Sheldenkar, Lertrak Srikitjakarn, Channé Suy Lan, Michael Wilkes, Terdsak Yano, Mark Smolinski

**Affiliations:** 1 Ending Pandemics San Francisco, CA United States; 2 Center for Invasive Species & Ecosystem Health University of Georgia Tifton, GA United States; 3 Food and Agriculture Organization of United Nations Rome Italy; 4 Department of Epidemiology and Biostatistics College of Public Health University of Arizona Tucson, AZ United States; 5 College of Extension University of Minnesota Rochester, MN United States; 6 Volte Health Systems Limited Abuja Nigeria; 7 Health Program Wildlife Conservation Society Bronx, NY United States; 8 EpiEcos LLC Flagstaff, AZ United States; 9 College of Public Health Department of Epidemiology and Biostatistics University of Arizona Tuscon, AZ United States; 10 Wee Kim Wee School of Communication & Information Nanyang Technological University Singapore Singapore; 11 Centre for Respiratory Diseases and Meningitis National Health Laboratory Service National Institute for Communicable Diseases Johannesburg South Africa; 12 SACIDS Foundation for One Health Morogoro United Republic of Tanzania; 13 Department of Population Health and Reproduction School of Veterinary Medicine University of California, Davis Davis, CA United States; 14 Institute for Scientific Interchange Turin Italy; 15 Open Dream Bangkok Thailand; 16 Saw Swee Hock School of Public Health National University of Singapore Singapore Singapore; 17 Boston Children's Hospital Boston, MA United States; 18 PODD Centre Chiang Mai University Chiang Mai Thailand; 19 Kawsang Co, LTD Phnom Penh Cambodia; 20 School of Medicine University of California, Davis Davis, CA United States; 21 Faculty of Veterinary Medicine Chiang Mai University Chiang Mai University Thailand

**Keywords:** participatory surveillance, One Health, citizen science, community-based surveillance, digital disease detection, environmental health, wildlife health, livestock health, human health, data standards

## Abstract

**Background:**

Emerging pathogens and zoonotic spillover highlight the need for One Health surveillance to detect outbreaks as early as possible. Participatory surveillance empowers communities to collect data at the source on the health of animals, people, and the environment. Technological advances increase the use and scope of these systems. This initiative sought to collate information from active participatory surveillance systems to better understand parameters collected across the One Health spectrum.

**Objective:**

This study aims to develop a compendium of One Health data parameters by examining participatory surveillance systems active in 2023. The expected outcomes of the compendium were to pinpoint specific parameters related to human, animal, and environmental health collected globally by participatory surveillance systems and to detail how each parameter is collected. The compendium was designed to help understand which parameters are currently collected and serve as a reference for future systems and for data standardization initiatives.

**Methods:**

Contacts associated with the 60 systems identified through the One Health Participatory Surveillance System Map were invited by email to provide specific data parameters, methodologies used for data collection, and parameter-specific considerations. Information was received from 38 (63%) active systems. Data were compiled into a searchable spreadsheet-based compendium organized into 5 sections: general, livestock, wildlife, environmental, and human parameters. An advisory group comprising experts in One Health participatory surveillance reviewed the collected parameters, refined the compendium structure, and contributed to the descriptive analysis.

**Results:**

A comprehensive compendium of data parameters from a diverse array of single-sector and multisector participatory surveillance systems was collated and reviewed. The compendium includes parameters from 38 systems used in Africa (n=3, 8%), Asia (n=9, 24%), Europe (n=12, 32%), Australia (n=3, 8%), and the Americas (n=12, 32%). Almost one-third of the systems (n=11, 29%) collect data across multiple sectors. Many (n=17, 45%) focus solely on human health. Variations in data collection techniques were observed for commonly used parameters, such as demographics and clinical signs or symptoms. Most human health systems collected parameters from a cohort of users tracking their own health over time, whereas many wildlife and environmental systems incorporated event-based parameters.

**Conclusions:**

Several participatory surveillance systems have already adopted a One Health approach, enhancing traditional surveillance by identifying shared health threats among animals, people, and the environment. The compendium reveals substantial variation in how parameters are collected, underscoring the need for further work in system interoperability and data standards to allow for timely data sharing across systems during outbreaks. Parameters collated from across the One Health spectrum represent a valuable resource for informing the development of future systems and identifying opportunities to expand existing systems for multisector surveillance.

## Introduction

### Background

In a world where anthropogenic landscape change drives zoonotic spillover, climate change exacerbates vulnerabilities in food security, and diseases further threaten biodiversity, effective and timely One Health surveillance mechanisms are imperative. The One Health United Nations Joint Action Plan (2022-2026) [1], Africa’s Centers for Disease Control Event-Based Surveillance (EBS) guidance [2], and World Health Organization’s “Defining Collaborative Surveillance” [3] underscore the critical need to integrate data across the animal, human, plant, and environmental health sectors. This paper examines the range of data parameters collected across the One Health spectrum through participatory surveillance methodologies.

### One Health Surveillance

The One Health approach recognizes the interdependencies of human, animal, and ecosystem health, aiming “to sustainably balance and optimize the health of people, animals, and ecosystems” [4]. Numerous national and subnational systems have undertaken initiatives for data sharing to develop One Health strategies, with support from organizations such as the United Nations, World Bank, and United States Agency for International Development (USAID) [5,6]. However, challenges remain in the implementation of One Health initiatives due to years of siloed professional training, budgets, data system development, data structures, and policies [7]. Rabies and influenza viruses have served as key drivers of multisectoral, collaborative surveillance and offer valuable models for advancing One Health approaches to address multiple pathogens. The need for the inclusion of plant health has more recently come to the forefront due to its critical role in food safety, food security, and ecosystem health [8].

Historically, traditional disease surveillance systems have largely been led by government entities and are often siloed, with a primary focus on human and livestock health. There is growing awareness of the need to harness more wildlife and environmental health event information to provide early warning of broader health threats facing both humans and animals. The Africa’s Centers for Disease Control EBS guidance emphasizes the integration of both formal and informal data sources, including hotlines and community-based EBS, to enhance outbreak detection [2]. To strengthen participatory surveillance systems, greater efforts are needed to capture data on putative drivers of pathogen spillover and disease emergence, including landscape change, environmental and climatic variables, and activities at the human-animal-environmental interface that bring animals and humans into contact with each other [9]. While previous literature has examined One Health tools and individual surveillance systems, no study to date has comprehensively documented the range of parameters collected in One Health participatory surveillance systems [7,10-12].

### Participatory Surveillance

Participatory surveillance involves a bidirectional process of receiving and transmitting data for actionable outcomes through the direct engagement of the target population [13]. This approach is increasingly used across multiple sectors to enhance early detection of and rapid response to emerging infections within a One Health framework. By engaging a wide variety of users including the general public, health care workers, rangers, farmers, and outdoor enthusiasts, participatory surveillance systems can provide early warning of potential outbreaks through crowdsourcing self-reported data in near real-time [14]. This active surveillance strategy empowers communities to act as the “eyes and ears” for detecting health threats at the interfaces where animals, humans, and environmental factors converge, creating opportunities for disease emergence.

Participatory surveillance systems play a crucial role in providing evidence-based feedback to users, enabling them to take timely actions to mitigate potential threats and facilitating connections with local resources for prevention and control. In the context of One Health, participatory surveillance extends beyond a human-centered lens to encompass plants, wildlife, livestock, and a multitude of environmental factors—habitats for disease vectors and water and air quality. The organizational structure of system users can vary widely, ranging from the general public to members of sector-specific associations or community alliances. For more structured community-liaison models, feedback is directed to the user group, empowering them to take action on behalf of the communities they serve.

Participatory surveillance has emerged as a powerful tool for enhancing traditional epidemiologic surveillance while fostering community engagement [15]. From reporting disease incidence to promoting mitigation behaviors, participatory surveillance has showcased its versatility across various infectious disease contexts, including influenza, cholera, COVID-19, and Zika [16-22]. One of the key benefits of participatory surveillance is its capacity to address and surmount many of the limitations inherent to traditional surveillance systems. In resource-constrained settings, conventional surveillance efforts often rely on overburdened health care and diagnostic systems and struggle to access difficult-to-reach communities, resulting in reporting delays or gaps. By empowering communities, participatory surveillance enables a proactive role in health monitoring, facilitating both prompt and comprehensive reporting of disease cases [23,24].

Ending Pandemics, a US nonprofit organization working to detect, verify, and contain outbreaks faster (and the supporter of the study presented here) developed the International Workshops on Participatory Surveillance (IWOPS), four of which have been held since 2012 in the United States, the Netherlands, Australia, and Cambodia [25]. IWOPS convened the creators, implementors, and key advocates to advance participatory surveillance approaches and strengthen partnerships among these systems. In 2016, the IWOPS community established a minimum dataset among systems collecting self-reported information to monitor the annual risk of influenza so that a common set of data standards could be developed. As a result, deidentified and disaggregated data are shared on a public platform, Global Flu View, for the purpose of improving situational awareness [26].

While Global Flu View represents one example of what is possible when participatory surveillance systems can “speak” to each other, data standards across the entire spectrum of parameters encompassing One Health are lacking. The One Health Participatory Surveillance Data Parameters Compendium (Multimedia Appendix 1) is a snapshot into the world of participatory surveillance for human, animal, and environmental health. The compendium approach was used as it not only illustrates the breadth and depth of what is being used in currently operating participatory surveillance systems across the globe, but it can serve to inform the needs of those contemplating the development of a participatory surveillance system or expansion of an existing system. As the compendium provides information on how data parameters are collected, it is poised to guide efforts to incorporate additional health sectors, adapt data parameters to align with other systems, and foster collaborations and data sharing. In the future, it could play a pivotal role in advancing data sharing, through informing the development of international data standards.

## Methods

### Development of Advisory Group

A multisector advisory group was assembled to guide the development of the compendium, support data collection, and provide cross-sectoral reviews encompassing animal, environmental, and public health sector perspectives. Selection criteria for advisory group members included expertise in participatory surveillance systems and integrating data from participatory systems into traditional surveillance systems. Subteams of experts in the areas of human, wildlife, livestock, and environmental health (including plants and vectors) were established. The experts represented a diverse range of public and government organizations, universities or research centers, nongovernmental organizations, and private sector entities. Geographically, they were affiliated with organizations in Asia (n=7), Africa (n=3), Europe (n=2), North America (n=10), and South America (n=2).

### Ethical Considerations

The study was reviewed by the University of Arizona Institutional Review Board and deemed not to involve human participants as defined by US Department of Health and Human Services and Food and Drug Administration regulations research (IRB protocol# 00002653). Within the body of the email, individuals were provided a disclosure statement. All participants were notified that they would receive a copy of the compendium, and their system would be listed in the acknowledgments. Data included in the compendium included terms and categories of parameters collected by systems and did not include any identifiable information. Participants were not compensated, and participation was voluntary.

### Data Collection

An invitation letter was emailed to a closed sample of representatives of 60 systems, using contact information from the One Health Participatory Surveillance map and 2022 landscape analysis [13]. Follow-up emails were sent twice, as needed, by members of the compendium advisory group to encourage responses from system representatives who did not reply initially. Emails described the goal for developing the One Health participatory surveillance compendium and requested respondents provide data parameters from their systems, methods for collection, and any special considerations for specific parameters using an attached template. The template had been used for parameter collection for IWOPS IV [27]. System representatives were offered the option to provide the requested information in other formats, such as a link to web-based systems allowing authors to independently collect relevant questions and parameters.

Data collected were in English. Parameters had already been collected for IWOPS IV in 2022 from: AfyaData, Cambodia 115 Hotline, EDDMapS or Wild Spotter, Forest First Pest Detector, Kidenga, MoBuzz, Outbreaks Near Me, One Health App Philippines, Participatory One Health Digital Disease Detection (PODD), SickSense/Open Dream, SURPRISE, World Animal Health Information System, SMART for Health, and WildAlert (formally known as the Wildlife Morbidity and Mortality Event Alert System) [27]. For these systems, emails were sent to confirm the parameters collected and to allow for revisions.

Out of the 60 systems contacted, 41 systems (68%) responded, and 3 systems (5%) responded they were no longer active and were not included in this analysis. Completed responses were received from 36/60 (60%) active systems; data were collected directly from publicly accessible apps or websites from 2/60 (3%) systems. 38 (63%) responses include the 9 country-level programs of InfluenzaNet. Seven of the 9 InfluenzaNet programs submitted 1 set of parameters and 2 (GrippeWeb and Hälsorapport) provided parameters individually.

### Data Analysis

The collected parameters were organized into 5 sections in the compendium: general, livestock, wildlife, environmental (including plants and vectors), and human health. Subcategories within each section were reviewed and refined through group discussions with the advisory group subteams specializing in livestock, wildlife, human, and environmental health. The focus of these discussions was to ensure usability, clarity, and accuracy. The human health advisory group subteam determined that parameters would be limited to those they determined directly relevant to infectious disease surveillance. The compendium focuses on parameters and does not describe structure, such as decision trees.

**Figure 1:**
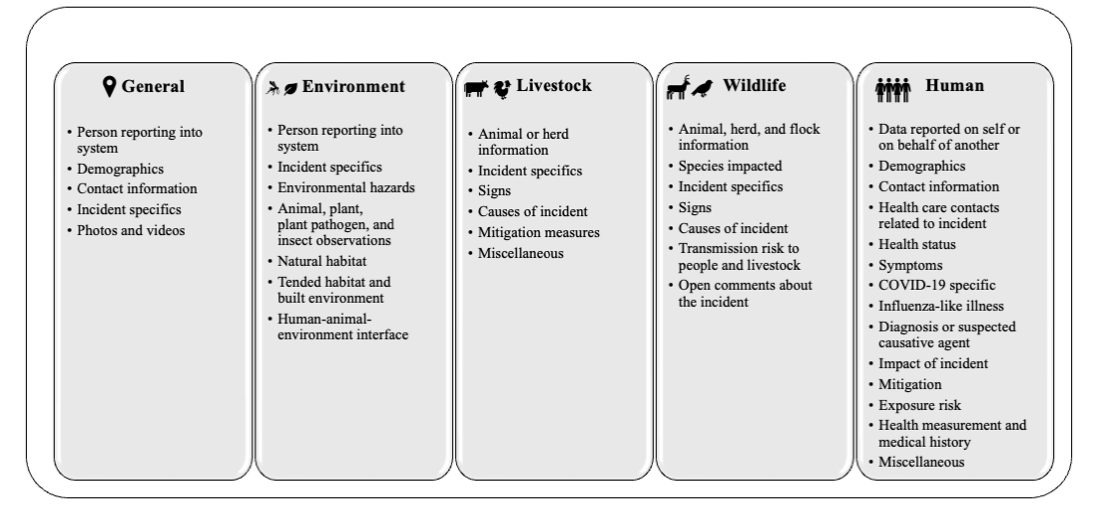
Categories of data parameters included in the 2023 One Health Participatory Surveillance Compendium.

Advisory group representatives reviewed the parameters and overall organization of the compendium and shared perspectives on key aspects to emphasize in the discussion and conclusion of this paper. Descriptive analyses were performed to examine the categories of parameters collected, the number and types of systems represented across sectors, and advisory group feedback on the parameters. Figure 1 outlines the compendium’s format, and the categories of data parameters collected.

## Results

### Participatory Surveillance Systems Represented in the Compendium

Data parameters were collected in English from across 38 participatory surveillance systems in Africa (n=3, 8%), Asia (n=9, 24%), Europe (n=12, 32%), Australia (n=3, 8%), and the Americas (n=12, 32%); only SMART for Health data were collected in several continents (Table 1). Approximately one-third of the systems (n=11, 29%) collect data from more than 1 sector, while the majority focus on a single sector. Many programs (n=17, 45%) are dedicated solely to human health, with fewer focusing exclusively on environmental or animal health (n=10, 26%). Most users contributing data to these systems are members of the general public (n=30, 79%), though other user groups include trained volunteers (n=6, 16%), wildlife rangers (n=2, 5%), farmers (n=2, 5%), wildlife rehabilitators and biologists (n=2, 5%), informal health care workers (n=1, 3%), and students (n=1, 3%). For example, SURPRISE is a system in which health care workers report on their own health, (n=1, 3%). Systems requesting weekly data reporting (n=16, 42%) primarily focus on human health, though some offer users the flexibility to determine the reporting frequency. Most of the remaining systems (n=20, 53%) allow users to set their reporting frequency.

**Table 1:** Description of global One Health participatory surveillance systems identified as active in 2023 including sectors included, location, user type, reporting frequency, and technology.

Systems			Sector		Countries		Type of user		Frequency of reporting		User technology
**AfyaData**			Animal, human, and environment		Tanzania, Mozambique		General public, trained volunteer, farmer, ranger, researchers, and students		Determined by user		SMS and mobile app
**Arizona Department of Fish and Game Hotline**			Animal and environment		United States		General public		Determined by user		Hotline and email or website
**Cambodia 115 Hotline**			Animal and human		Cambodia		General public and health officer		Weekly, determined by user		Hotline
**California West Nile Bird Reporting**			Animal		United States		General public		Determined by user		Hotline and email or website
**Colab**			Animal, human, and environment		Brazil		General public		Determined by user		Mobile app, hotline, and email or website
**CoughWatch SA**			Human		South Africa		General public		Weekly		Email or website
**DisApp**			Environment		India		General public		Determined by user		Mobile app
**EDDMapS/Wild Spotter**			Animal and environment		United States and Canada		General public and trained volunteer		Determined by user		Mobile app and email or website
**eWHIS^a^**			Animal and environment		Australia		Trained volunteer		Monthly, determined by user		Hotline and email or website
**FluTracking Australia**			Human		Australia		General public		Weekly		Email or website
**FluTracking HongKong**			Human		Hong Kong		General public		Weekly		Email or website
**Forest Pest First Detector**			Environment		United States		General public		Determined by user		Mobile app and email or website
**Guardians of Health**			Human		Brazil		General public		Daily		Mobile app
**Garden Wildlife Health Project**			Animal		Great Britain		General public		Determined by user		Email or website
**iMammalia**			Animal		Europe		General public		Daily		Mobile app and email or website
**InfluenzaNet programs**											
	FluSurvey.net	Human		United Kingdom		General public		Weekly		Email or website	
	Grippenet.ch	Human		Switzerland		General public		Weekly		Email or website	
	Grippenet.fr	Human		France		General public		Weekly		Email or website	
	GrippeWeb	Human		Germany		General public		Weekly		Email or website	
	Hälsorapport, Health Report Sweden	Human		Sweden		General public		Weekly		Email or website	
	Infectieradar.be	Human		Belgium		General public		Weekly		Email or website	
	Infectieradar.nl	Human		Netherlands		General public		Weekly		Email or website	
	Influmeter.dk	Human		Denmark		General public		Weekly		Email or website	
	InfluWeb	Human		Italy		General public		Weekly		Email or website	
**I-TICK**			Environment		United States		General public		Determined by user		Mobile app
**Kidenga**			Environment and human		United States and Puerto Rico		General public		Weekly		Mobile app
**MoBuzz+**			Environment and human		Sri Lanka		General public, health authorities		Determined by user		Mobile app and email or website
**MosApp**			Environment		India		Formal, informal health care workers		Determined by user		Mobile app
**Outbreaks Near Me**			Human		United States, Canada, Mexico		General public		Weekly, Determined by User		SMS and email or website
**PODD^b^**			Animal, human, and environment		Thailand		General public, trained volunteer, farmer, and local government		Fortnightly		Mobile app
**SMART for Health**			Animal and environment		Laos, Cambodia, Vietnam, and Guatemala		Rangers		Determined by user		Mobile app
**SickSense or Sabaidee**			Human		Thailand		General public		Daily, Weekly, Determined by user		Mobile app
**SURPRISE+**			Human		Switzerland		Health care workers		Adaptive according to epidemiology		SMS and email or website
**Tamil Nadu Population Health Registry**			Human		India		Trained volunteer		Daily		Mobile app and paper
**Tick Tag Go**			Environment		United States		General public		Determined by user		Mobile app and email or website
**Weedspotters Australia**			Environment		Australia		Trained volunteer		Determined by user		Mobile app
**Wild Health Net Ebola**			Animal and environment		Republic of Congo		Wildlife biologists		Determined by user		SMS, mobile app, and email or website
**WildAlert**			Animal		United States		Wildlife rehabilitators, state wildlife agencies, and university researchers		Weekly, determined by user		Email or website

^a^eWHIS: electronic wildlife health information system.

^b^PODD: participatory one health digital disease detection.

### General Parameters

General parameters include those not specific to any sector—such as user demographics and contact information, incident location, and options for photo or video uploads. Systems with regular users often collect user demographics and contact information during the initial login, eliminating the need for repeated entry on subsequent logins. Substantial variation exists on how demographic data are collected—particularly as related to gender, ethnicity, education, and profession. In contrast, contact information, such as email and phone numbers, are collected uniformly across most systems. Incident location is commonly collected using automated geolocation. Additionally, some systems include mechanisms for photo or video upload to provide supplementary information.

### Environmental Health Parameters

#### Overview

The range of parameters collected related to environmental health is as broad as the field itself—encompassing activities, such as tracking logging, monitoring water contamination, and detecting invasive species and disease vectors. Over half of the systems (8/15, 53%) collecting environmental health data also include a focus on animal and human health. Kidenga collects data on environmental factors including the risk of mosquito exposure alongside human health symptoms. SMART for Health captures key environmental and epidemiological features as well as information on wildlife and livestock. Participatory surveillance systems vary in scale, with some designed for local, relatively small geographic areas and others operating on global platforms, such as iNaturalist. Several vector-focused systems reported that weather information, such as rainfall and temperature, can be added retrospectively by linking it to date, time, and location. Natural habitat data in these systems includes landscape descriptions, while details of tended habitats include descriptions of yards, gardens, and ponds. The built environment is also considered, with systems collecting data on housing structures, water and sanitation, and energy sources, such as fuel and electricity.

#### Plants

Three systems collect data on plants, including the Early Detection and Distribution Mapping System, which also gathers data on animals. Collected parameters include date or season, high-resolution digital images, information on the reporter, and detailed location information. Date, time, and GPS coordinates are often extracted directly from digital image metadata and autofilled in the report. Other details include ecosystem type, whether the area is tended or untended, seasonality, and additional species, such as host or insect pests.

Species identification is commonly performed by the primary reporter, with photos allowing for additional verification. Species identification is done in 1 reporting field or across multiple fields, capturing details or images of features such as flowers, seeds, leaves, leaf arrangements, and the entire plant. All the plant-focused systems record data on invasive plants and some databases also include information on legal, noxious weed status. High-quality, high-resolution digital images and tightly calibrated geographic locations are noted to be particularly important in these systems.

#### Vectors

Almost half (7/15, 46.7%) of the environmental systems are explicitly designed to monitor vectors. Notably, the Early Detection and Distribution Mapping System amalgamates vector, animal, and plant surveillance. Among the 7 vector surveillance systems that primarily focus on the environmental sector, 3 exhibit a multisectoral approach. Kidenga and MoBuzz+ bridge the environmental and human domains.

Vector exposure is collected in several ways across systems including incident-specific data with details on the date and precise location of the vector bite incident, specific vector bite information, and the detection of vectors in the vicinity. Often, observations include the perceived number of vectors and their morphology.

Data encompasses information on any formal diagnoses of vector-borne diseases and community prevalence of disease. Vector identification data incorporates visual documentation using photos uploaded by the user. Photos enable experts receiving the data to visually identify and assess vector size, color, and, where possible, species. Vector habitat data includes breeding sites and the detection of larvae, which use the number of measurement containers at a given location and whether they contain larvae. A number of systems integrate the history of recent outdoor activities to better understand the potential exposure of vectors. Vector mitigation data collects information on the use of pesticides, as well as any treatments for controlling or countering vector exposure, and appropriate clothing.

### Animal Health Parameters

#### Wildlife Health

Nine of the participatory surveillance systems collect data on wild animals. SMART for Health includes parameters to record the health of free-ranging livestock that may be present in protected natural habitats. Most of these systems were developed with the goal of syndromic wildlife disease surveillance except for 2 systems— one that was designed to collect data on geographic distribution and abundance of wild mammalian species and another that focuses on early detection of invasive wildlife species [13]. Among these 9 systems, 4 (44%) also collect information on a range of environmental variables. Many variables focus on risk factors or environmental drivers of adverse health events/disease outbreaks in animals as well as humans such as environmental disturbance, or interactions at the wildlife-livestock-human interface. In addition, WildHealthNet—Ebola integrates data on both wildlife and human health parameters specifically for Ebola Virus Disease surveillance.

Common across most of these systems are parameters specific to wildlife incidents, including species or taxa groups of wild animals involved in the event, date, and numbers and location of affected animals. For location information, a few of the systems have the capability to capture specific GPS coordinates as the user digitally enters data or to generate GPS coordinates from an address through geocoding. Typically, systems also include data on the condition of the animal(s) involved in the incident, including abnormal findings in sick, injured, or dead wild animals and a presenting syndrome or clinical classification of these animals. Depending on the system, data are reported at the level of the individual animal or species.

Wildlife health parameters are collected in a variety of formats including dropdown menus, check boxes, and fields with free text. Categorizing free text data for surveillance purposes through natural language processing models has been applied by WildAlert to categorize data for surveillance purposes. Collection of data related to risk of exposure to humans—such as handling of sick wildlife or bats roosting near a home was also included.

Two of the systems also collect data on definitive diagnosis. For systems that integrate clinical wildlife data, this data, if available, is included for both individual animals as well as events. Other parameters among these systems include circumstances surrounding the incident and the potential causes of illness, injury, or death at both individual and species levels. Furthermore, one system also allows the user to enter data on the estimated population size and number of animals at risk for wildlife incidents enabling estimations of disease occurrence among a population.

#### Livestock Health

Only 4 surveillance systems collect data on livestock, and all of them are multisectoral. These systems together cover a wide variety of domestic species including livestock, poultry, dogs, and cats. These systems collect data using a range of technologies including calls and mobile apps. They focus on common parameters such as species, age, sex, and location of affected animals, including the magnitude of morbidity and mortality at individual and group (herd or flock) levels. Clinical signs of illness were either reported by body systems (checkboxes) or as free text. AfyaData collects health information on the owner as well as on the animals. Only 2 systems include parameters related to companion animals.

### Human Health Parameters

Data parameters on human health are collected by 23/38 (61%) systems, with the majority (17/23, 74%) collecting only human health data. Of the 6 collecting multisector data, 3 systems collect data parameters across all One Health sectors, that is, human, animal, and environmental health.

The data parameters for human health cover virtually every organ system within the human body. Across all the systems monitoring human health, fever-related terms are the most common set of data parameters for reporting symptoms of illness. Additional symptoms indicative of influenza-like illness (ILI), including headache, sore throat, and cough are also among the most common symptom parameters collected. Gastrointestinal symptoms are the next most common set of data parameters across the systems collecting human health data.

In 2020, COVID-19-specific symptoms were added to several of the systems, many of which already collected ILI symptoms to track influenza, but now added “loss of taste or smell,” for example. COVID-19 and influenza diagnostic testing and vaccination history are common among the systems monitoring human health only.

Outbreaks Near Me asks the user to report being “healthy.” Only when the user reports not being “healthy” do they receive queries on symptoms, events, or other options available within a specific system. Most systems focus on symptoms of illness and the onset of the timing of the symptoms. Several systems ask about health-seeking behavior and the type of health care system visited. Kidenga asks about the health status of others in the household and others inquire about the number of people in the household.

Absenteeism from work or school or changes in daily routine due to illness is captured by many of the systems. Common parameters also include various risk factors, such as exposure to other people with similar symptoms, exposure to pets, work exposure, and recent travel history. Furthermore, a few of the systems collect data on preexisting medical conditions as well as lifestyle parameters such as exercise, smoking, and alcohol consumption.

## Discussion

### Overview

The One Health Participatory Surveillance Data Parameters Compendium showcases the breadth and depth of how systems are using community engagement to collect multisector data to better inform shared threats. This resource highlights opportunities to expand One Health surveillance and identifies parameters currently collected across multisector systems. It encompasses a broad array of data modes and mechanisms used for data collection, addressing challenges with optimizing user time for data entry and improving accuracy. Additionally, the compendium reveals sector-specific differences in how and when data are collected.

### Principal Findings

#### Opportunities and Challenges for System Augmentation or Development

Only 3 systems—PODD, AfyaData, and Colab—include data from all 3 sectors. For existing single-sector surveillance systems, the compendium offers a framework to identify key parameters for expanding into this type of multisector, One Health surveillance system. For example, wildlife-focused systems could incorporate data on the surrounding environment or on the health of humans interacting with wildlife. Similarly, human health–focused systems in urban areas could expand to include data parameters on the built environment, proximal wildlife, and the health of companion animals. In rural areas, human health systems could include parameters on the health of livestock and wildlife within the shared environment. Given the increasing incidence of emerging infections, data on drivers of disease emergence—such as environmental and weather changes—are valuable contributions to participatory surveillance systems [7]. With the interconnectedness of human, animal, and environmental health, the expansion of environmental systems to include these components is a logical progression.

Most participatory systems are community-driven and developed; thus, parameters are added to a given system to address specific community needs and concerns. Therefore, piloting terms and questions within a target population is essential to ensure wording and translation are culturally appropriate and that the requested parameters are accurately captured. Ensuring integration with governmental surveillance efforts across multiple ministries and departments is also critical when expanding beyond one sector to support more sustainable and successfully coordinated One Health approaches.

Expanding a surveillance system to collect parameters across sectors requires consideration of the time users are willing to invest. Users can only reasonably be expected to spend a limited amount of time answering questions or selecting from menu options. Many systems address this by incorporating automatic data inputs, such as timestamps or geolocation, to save time and improve accuracy. Decision-tree frameworks are also used to streamline data collection and gather targeted information; for example, a “yes” response to a question about “cough” could lead to a follow-up inquiry about “bloody cough.” Wildlife and vector programs frequently rely on images to minimize the number of questions and to ensure accurate species identification.

Advancements in technology allow for automated data collection, such as integrating ambient temperature measurements, which can enhance user experience, save time, and improve data quality. Variations in education and literacy levels within communities must be considered. Working closely with the community during system development can help address these challenges and foster user engagement.

Existing siloes among One Health sectors can limit the effective use of data and hinder cross-sectoral collaboration. Existing system infrastructure as well as sensitivities around data privacy can create barriers that make it difficult to collect and share parameters across sectors. Current systems also operate at varying spatial scales. For example, human health data is often protected by privacy regulations, making it unethical to include precise geocoordinates. On the other hand, some environmental parameters, such as mosquito habitats or locations of dead wildlife, require specific geocoded data to enable required actions. Data privacy is critical and can be addressed through user-specific access layers. Integrated dashboards and similar tools can enable health authorities from multiple sectors to visually identify disease hotspots, pinpoint areas with elevated disease transmission risk, and prioritize mitigation efforts. For instance, in Mo-Buzz+, public health inspectors can review reports on communal dengue cases, identify mosquito breeding sites, and detect community practices that contribute to mosquito breeding. To protect privacy, this type of information could be aggregated to a neighborhood level before being shared with public users, preserving anonymity while providing actional insights.

For livestock and plant health, additional sensitivities regarding the location of incidents may arise due to potential economic impacts. Systems address these concerns in various ways, such as obscuring publicly available information and anonymizing reporter details. Any system handling sensitive information must proactively address these concerns to ensure confidentiality and minimize potential risks.

#### System Structure and Timing Impact Types of Information Gathered

The types of information gathered by surveillance systems vary based on their structure. Systems designed to collect longitudinal data often include more “general” parameters during the initial registration process, reducing the need to recollect this information during subsequent logins. This approach saves the user time by focusing additional questions only on cases where the user reports “unhealthy” conditions for that week. Collecting longitudinal data from a known population allows these systems to calculate both numerators and denominators for analyzing trends and spatial-temporal patterns. Visualizing reports over time and space strengthens disease tracking and anomaly detection. This approach is most common for human health systems, as well as Garden Wildlife Health and WildAlert. In SMART for Health, users (wildlife rangers) track animal populations over time, allowing for the identification of both population and disease trends.

Some systems that collect data on wildlife and environmental factors rely on users to report opportunistically when they encounter a specific animal or setting. As these encounters may be the only opportunity to collect data on the health event, extensive data may be needed. To make the process user-friendly, these systems often incorporate photographs and automated downloads of data such as geolocation and limit the collection of extensive user and animal demographic details. While one-time reporting for plant or environmental health incidents is highly valuable for enabling prompt action, it rarely supports longitudinal surveillance or trend detection. Identification of ways to expand follow-up reporting by users may be appropriate, particularly in the context of suspected outbreaks. Systems collecting data on human-animal interactions, such as bat exposures, are especially valuable for surveillance initiatives aiming to improve understanding of the underlying factors driving pathogen spillover and disease emergence.

Dropdown menus offer the advantage of standardizing information, which facilitates data recording in the field, promotes consistent data analysis across units, and supports data integration across systems. In contrast, free text data fields capture a wide array of information from the user or when the user may not have the technical background or expertise to properly categorize a case.

Language and cultural barriers within communities can result in disparities in data capture. Addressing these challenges requires strategies such as raising community awareness and education to enhance disease literacy and improve access to health care. Using tailored engagement approaches for specific population segments and fostering collaboration with local stakeholders is essential for improving surveillance and response outcomes.

#### Data Standards

Improving interoperability and data sharing among participatory surveillance systems can allow for early incident detection. Improving interoperability (the ability of different systems to exchange, interpret, and use data accurately) and data sharing on a regional or global scale will require the development of data standards. Data standards facilitate international data exchange and better collaboration. A data standard is an agreed-upon approach, to allow for consistent measurement, qualification or exchange of an object, process, or unit of information. Data standards include agreements on several topic areas including representation, format, definition, structure, use, documentation, and management of data [28,29]. They enable transparency and understanding, and the use of standards promotes common, clear meanings for data [29]. Several systems in this study collect similar categories of parameters but use different questions or methods to gather the information. This variability is often necessary to account for community-specific differences, such as how questions about ethnic groups are phrased. Ideally, while user interfaces may differ to accommodate these variations, the backend systems should be designed to integrate the collected information.

### Sector-Specific Findings

#### Human Health

It is not surprising to see fever as the most prevalent symptom among systems designed to detect early infection in humans. Many have noted that fever monitored on an ongoing basis with temporal and geospatial visualization could be an early indicator of a communicable disease threat [30,31]. Since respiratory diseases have the highest probability of causing local outbreaks, regional epidemics, and potentially a pandemic, many systems monitor for ILI and this likely accounts for the frequency of ILI-terms among the systems. Several systems collect parameters to complement symptom data reported—such as health care–seeking behavior to help track the severity of illness or travel to help understand exposure risks.

#### Environmental Health

The inclusion of environmental parameters in multisector systems allows systems to understand the complete context in which an incident occurs. Identification of potential vector-breeding locations, water contamination, and habitat change may allow for intervention before the spread of disease into local animal and human populations. Environmental parameters also capture how animals and people may be impacting the health of the environment.

Like wildlife health, environmental health programs often rely on event-based reporting. For example, a person enters information about vector exposure, seeing a contaminated location, or spotting a presumed invasive species. Automated features, such as automated collection of climate parameters, geolocation, and image uploads, allow the system to accurately capture a situation without solely relying on an observer’s subjective reporting. To help standardize data, systems use tools such as iNaturalist, which may include predefined choices, and have internal experts reviewing photographs to correctly identify vector, animal, and plant species. The collection of incident-specific details is foundational for understanding the spatiotemporal distribution of vectors and their potential role in disease transmission.

Despite the comprehensive parameters used, the underreporting of vector-borne disease cases in humans remains a persistent concern. This phenomenon is especially prevalent in regions with limited access to health care services or where vector-borne diseases are poorly understood [30,31].

#### Livestock and Wildlife Health

Relatively fewer participatory systems exist for livestock compared to systems dedicated to other sectors. Participatory epidemiology is often used to address specific outbreaks in livestock health [32]; ongoing participatory surveillance systems may not be as common due to heavy reliance on governmental surveillance programs for outbreak detection. In the compendium, livestock parameters are from systems also collecting data on wildlife, people, or the environment, reflecting the interconnectedness of livestock among each of the other sectors. Given the daily proximity of pets to owners and the potential for bidirectional transmission, the expansion of participatory surveillance to inform companion animal outbreak detection is needed.

Similar to environmental health systems, wildlife health systems are often event-based—increasing situational awareness of disease occurrence and informing on common disease threats in real time. Information stemming from these systems are commonly underused as resource for monitoring long-term trends in population health and mostly are untapped as resource by sectors outside of wildlife health.

Across wildlife participatory surveillance systems, data accuracy remains a significant challenge, as users often face difficulties accurately identifying wildlife species and describing abnormalities in sick, injured, or dead wild animals. Additionally, the integration of disparate data sources and achieving interoperability between systems is limited due to the diverse types and formats of data collected. The lack of harmonized data in wildlife health surveillance has been a long-standing issue; however, systems designed for large, multilingual, and strategically diverse user bases hold promise for reversing this trend.

The increasing use of machine learning methods, such as natural language processing, offers new opportunities for categorizing data, and enabling comparisons across multiple surveillance streams and systems [33,34]. However, these approaches require compatible language inputs or effective translation mechanisms. The compendium highlights the value of collecting and analyzing prediagnostic data during incidents, which facilitates the rapid detection of clusters of animals presenting with similar abnormalities that might signify a disease outbreak or other adverse health event in wildlife.

### Comparison With Prior Work

This work builds upon the landscape analysis of participatory surveillance programs and IWOPS workshops reviewing data parameters and working toward data standards [13,25]. Prior publications have highlighted frameworks and steps for building One Health surveillance systems, including methods for better cross-sectoral stakeholder engagement and ways to overcome implementation barriers [7,10,11]. Identified barriers to the implementation of One Health surveillance systems include incompatible vocabularies, professional divisions, isolated datasets, and the lack of data standards. When developing new One Health participatory surveillance systems, this compendium provides a concrete starting point with real-world examples to facilitate dialogue on data elements and standards for sharing across siloed sectors. A critical analysis by Behravesh et al [12] of landscaping globally available One Health tools uncovered 50 tools that advance One Health. The results highlight that most of these tools addressed assessment and few addressed planning and prioritization. This compendium provides a practical One Health tool to facilitate and support planning, prioritization discussions, and One Health participatory surveillance system design. Prior studies have noted the value of participatory surveillance in war-affected camps and the use of One Health surveillance to capture the impact of the environment on refugee health [35,36]. Interactions between humans and animals are often ignored with regard to the health of refugees, but animal populations are often closely tied to refugee communities. This compendium can be applied to improve One Health disease surveillance for displaced populations in high-risk regions.

### Limitations

Data parameters included in the compendium reflect a point in time and are not fully representative of the entire field of participatory surveillance. The parameters were collected in English, through an English-language survey, which may have limited participation from non-English systems. Furthermore, selection bias could be present, as systems were identified based on the participatory surveillance map. Additional validation of data parameters was not conducted because many systems are not publicly available; the parameters included in the compendium are based on the information directly provided by the systems.

Most systems use a decision-tree framework to structure their questions. The compendium does not include this format, focusing on parameters over the structural design. To partially address this limitation, the “special considerations” column in the compendium provides comments on how specific parameters relate to others. Future iterations of the compendium should explore methods to illustrate system structures, providing context for how parameters are used within their frameworks.

Given the compendium’s focus on outbreak detection, parameters related to chronic disease were not included. Future exploration on how mental health, chronic disease, toxic exposures, and other non-outbreak–related parameters are collected would be informative for participatory surveillance systems aiming to capture a broader set of health parameters.

### Conclusions

Participatory surveillance enhances traditional means of health surveillance by empowering communities to identify threats at their source. The compendium highlights how existing participatory surveillance systems are already incorporating a One Health approach. Expanding opportunities for multisector data collection through these participatory systems aligns with recent guidance from the United Nations Quadripartite [3,37]. Additional research is required to inform international data standards for One Health participatory surveillance. Pilot studies could provide useful mechanisms to capture various multisectoral data collection and sharing techniques. Furthermore, ethical issues of data privacy are always essential to consider when capturing personal information. Participatory surveillance provides critical surveillance data that can be deidentified and collated at a specific geographic level, which facilitates anonymity of the responses. The compendium offers a reference to strengthen community capabilities for developing multisector surveillance systems to inform early detection of health incidents threatening themselves, their families, and the environment. Decision makers and practitioners can use the compendium as a tool to build a new participatory surveillance system or augment their existing systems to include a One Health approach. The compendium highlights the need to work toward system interoperability and data standards to enable community-level surveillance to have national, regional, or global implications in outbreak detection and response.

## Appendix

Multimedia Appendix 1 One Health Participatory Surveillance Compendium.

## Abbreviations

EBS: event-based surveillance
ILI: influenza-like illness
IWOPS: International Workshops on Participatory Surveillance
PODD: Participatory One Health Digital Disease Detection
USAID: United States Agency for International Development

## Data Availability

All data generated or analyzed during this study are included in this published article [and its supplementary information files—Multimedia Appendix 1].
